# The Compensatory Role of Diverse Workplaces: Parental Workplace Educational Composition and Children's Higher Education Enrolment

**DOI:** 10.1111/1468-4446.70013

**Published:** 2025-08-11

**Authors:** Laura Heiskala, Margus Pruel

**Affiliations:** ^1^ INVEST Research Flagship Center University of Turku Turku Finland

**Keywords:** compensation, educational transition, higher education, social origin, weak ties, workplace

## Abstract

Studies consistently find family background differences in educational attainment, with parental education being an important factor in families' educational decision‐making processes. Alongside parents’ own resources and accomplishments, research has shown that both immaterial and material resources from extrafamilial connections, such as extended family members, are positively associated with children's educational attainment and may compensate for a lack of resources within the immediate family. In this study, we examine the compensatory role of parental workplace ties in shaping children's educational choices. Using full population register data from Finland, we find that children from lower‐educated families are more likely to enrol in higher education if they have a parent working among highly educated colleagues. We discuss the importance of diverse environments for educational mobility and aim to shed new light on the role of weak ties in educational decision‐making.

## 1 Introduction

Socioeconomic segregation in schools, workplaces, and neighbourhoods is becoming increasingly prevalent, creating social spaces where individuals from different socioeconomic groups rarely interact (Mijs and Roe 2021). This trend has concerning consequences, including a diminished understanding of the lives of other social classes and reduced economic mobility (e.g. Chetty et al. 2022a, 2022b). When people do not interact with others from different social classes, it can have troubling consequences for social cohesion and even for their understanding of the extent of inequality within society (Mijs and Usmani 2024).

Diverse social spaces seem to be beneficial especially for those from lower socioeconomic backgrounds. Research on “neighbourhood effects” highlights that socioeconomically heterogeneous neighbourhoods are particularly advantageous for children from low socioeconomic backgrounds, enhancing their educational outcomes and social mobility (e.g. Patacchini and Zenou 2011; Chetty and Hendren 2018). Heterogeneous school composition net of neighbourhood effects (Kauppinen 2008), and educationally heterogeneous workplace environment (Baranowska‐Rataj et al. 2023), have also been shown to level out differences, in educational choices and intragenerational mobility, between different social classes.

In this study, we focus on workplaces. While workplaces are generally segregated environments (e.g., Avent‐Holt and Tomaskovic‐Devey 2019; DiTomaso et al. 2007; Reskin et al. 1999), they can also serve as avenues for individuals to pursue social mobility and career advancement (K.‐H. Lin et al. 2025). Individuals in workplaces are subject to divisions of labour that are both technical and social, such as departments and jobs, and are making claims on organizational resources like earnings and promotions, but also on interactional and experiential resources like autonomy and respect (Avent‐Holt and Tomaskovic‐Devey 2019). Workers value positive social relations in their workplace, and closed workplace networks can increase shared norms and solidarity within the workplace community, which can even boost their future employment prospects (Henriksen et al. 2025).

Our aim is to explore workplace educational diversity and its contribution to building intergenerational educational mobility. Specifically, we study whether having a parent exposed to highly educated co‐workers can help reducing the social origin gap in higher education enrolment. Educational choices form a major mechanism of intergenerational class reproduction and are also important tools for facilitating class mobility (Blau and Duncan 1967). Extensive number of studies show that family background and parental resources, such as parental educational level, are associated with children's educational outcomes (e.g. Shavit and Blossfeld 1993; Pfeffer 2008; Breen et al. 2009; Jackson 2013). Furthermore, studies have shown that extrafamilial resources, such as those from extended family members, can compensate for a lack of resources within the immediate family and thus reduce the social origin gap in educational attainment (Jæger 2012; Erola and Kilpi‐Jakonen 2017; Erola et al. 2018; Lehti et al. 2019). Our contribution to this literature is to focus on non‐kin relationships, specifically co‐worker networks, which, despite their increasing level of segregation, are more likely to be heterogeneous and diverse compared to kin‐based connections.

Certain aspects of the workplace and their social networks make them worth considering in this respect. Overall, most adults spend a significant part of their day at their workplaces and most adult non‐kin relationships are formed at workplaces (Marks 1994). Moreover, workplaces are social spaces in which all individuals form ties irrespective of social class, in contrast to neighbourhoods, where those from lower socioeconomic strata are more likely to form ties, and colleges, where those from higher socioeconomic strata are more likely to make friends (Chetty et al. 2022b). We draw from Granovetter's (1973) well‐known thesis about the “strength of the weak ties” which states that acquaintances, referring to connections to workmates or other people who are not family members or close friends, are crucial for information flows. Weak ties often connect people hailing from different social groups; this allows them to gain access to different kinds of information (Granovetter 1973). Connections to highly educated colleagues have been shown to be important for less educated employees, as they can help them build intragenerational social mobility, referring to mobility from one position to another within one's lifespan (Baranowska‐Rataj et al. 2023; Chetty et al. 2022a; Lin et al. 1981). We study their implications for intergenerational inequality.

We study these questions in the Finnish context using the high‐quality Finnish full population register data. Our analyses, which use the 1989–1993 birth cohorts, show that children with lower‐educated parents tend to enrol in higher education more often if their parents work around highly educated co‐workers compared to other children with lower‐educated parents. In the following sections, we discuss the literature concerning families' educational decision making and the importance of strong and weak ties in this process, after which we introduce the study context and discuss our results.

## 2 Theoretical Background

Numerous studies demonstrate that there is a strong correlation between family background and educational attainment, even in highly egalitarian contexts (e.g. Shavit and Blossfeld 1993; Shavit et al. 2007; Pfeffer 2008; Breen et al. 2009; Jackson 2013). Since educational choices are usually made at a relatively young age, parental resources play an important role in the educational decision‐making process. The unequal distribution of various kinds of resources between families (e.g. material, cultural, or genetic resources) leads to differences in children's educational performance. Thus, children of privileged origins tend to perform better at school (Jackson 2013; Boudon 1974).

These social background differences in educational performance can partly explain why educational levels are transmitted from one generation to the next. However, research has shown that children from privileged backgrounds tend to continue their educational pathways further and choose more prestigious tracks even after differences in educational performance have been considered. Leaning on the rational choice framework, the educational decision‐making model developed by Breen and Goldthorpe (1997) states that there is a strong motivation to avoid downward mobility within families, which influences educational choices. This model has three main components that individuals consider when evaluating their choices while simultaneously avoiding intergenerational downward mobility: the cost of education, the benefits of education, and probability of success in their studies (Breen and Goldthorpe 1997). Families are motivated to avoid downward social mobility across generations, and as a decision‐making unit, they make educational choices that aim to secure children's position in the same social class as that of the parents.

However, the educational choices are not always rational and may be affected by many biases. Individuals use available information in a restricted manner, limiting their attention to a small group of options and leading to both over‐ and under‐estimation of risks, benefits, skills, and abilities (Barone et al. 2017). In addition to these heuristics, decision‐making units, namely families, often lack full and accurate information on available educational options and the outcomes of different pathways (Barone et al. 2017; Kerr et al. 2020). These information barriers regarding higher education limit families' informed decision‐making in a socially stratified manner (Abbiati and Barone 2017; Forster and van de Werfhorst 2020).

Various intervention studies have explored whether the social origin gap in higher education access can be reduced by breaking down these information barriers, most often regarding the understanding of the actual costs and benefits of education (e.g. Barone et al. 2017; Ehlert et al. 2017; Abbiati et al. 2018). The results of these intervention studies have been mixed thus far, with some of them showing that breaking these barriers can reduce social inequalities in access to higher education, especially if these interventions include assistance or individual guidance (Herbaut and Geven 2020). Fewer studies have examined how information interventions on parents can reduce social origin differences in higher education enrolment (Bergman 2019). Regarding parents, Barone and colleagues (2018) demonstrate that in the context of upper secondary education, when parents are better informed about the actual risks associated with different educational options, the social origin gap in choosing academic tracks is reduced.

In addition to interventions, new information can also be accessed via other channels—for example, through social networks. Besides personal resources, individuals are embedded in social networks that provide various social resources accessible through social actions (N. Lin 1999, 2001). These social resources may compensate for or reinforce any inequalities stemming from the families' own pool of resources (Erola and Kilpi‐Jakonen 2017). Studies have shown how, for example, highly educated extended family members are positively associated with children's educational attainment of low social origin families, and thus compensate for the lack of resources in the immediate family (Jæger 2012; Erola et al. 2018; Lehti et al. 2019). This implies that connections to highly educated adults may reduce socioeconomic differences in children's educational attainment in lower‐educated families.

Close friends and relatives, however, often belong to very similar social circles and share similar educational levels with immediate family members. This limits their potential in breaking down information barriers and providing new perspectives. In addition to the social circles formed by family, relatives, and friends, individuals are embedded in other social contexts such as neighbourhoods and workplaces.

Despite being exposed to a variety of individuals in these social settings, individuals may not have close connections and strong ties with all of them. However, individuals may know lots of co‐workers or neighbours somehow. In his thesis on the 'strength of weak ties,' Granovetter (1973) argued that weak ties, as opposed to strong ties like kinships or close friendships, play a crucial role in information flow and mobility. This is because they connect individuals with others who may not share much in common, thereby forming bridges for information exchange across diverse social groups. Hence, the strengths of weak ties include making a large number of connections and accessing new kinds of resources and information. Connections between co‐workers are the most commonly used example of weak ties. Previous studies have shown the applicability of co‐worker weak ties on, for example, information flows regarding new job opportunities (Lin et al. 1981) and building mobility inside the company (Podolny and Baron 1997).

In general, people tend to form ties with people who are similar to themselves (McPherson et al. 2001). However, just being exposed to different kinds of people boosts interaction among social groups (Chetty et al. 2022b). Exposure to individuals from different social classes, such as sharing classrooms, explains half of the effect of economic connectedness–that is, whether individuals have friends from a different socioeconomic group (Chetty et al. 2022b).

Although workplaces are often segregated organizations in which different forms of stratification take place (e.g. Avent‐Holt and Tomaskovic‐Devey 2019; DiTomaso et al. 2007; Reskin et al. 1999), in workplaces, employees may interact with people with whom they might otherwise not form connections. Workplace networks and co‐workers often include individuals with compensatory and interdependent skills (Neffke 2019). This, in turn, leads to teams of co‐workers that are only partially similar and, in some cases, could also differ substantially depending on the skills required and how close these complementary skills are to each other. These heterogeneous workplace networks seem to be especially beneficial for those from lower social origins. Weak ties benefit lower‐class individuals in their career mobility when they have access to information from higher‐class individuals (Lin et al 1981). More recently, Baranowska‐Rataj et al. (2023), using Swedish register data, showed that connections to highly educated co‐workers help less educated workers escape low‐wage employment.

**FIGURE 1:**
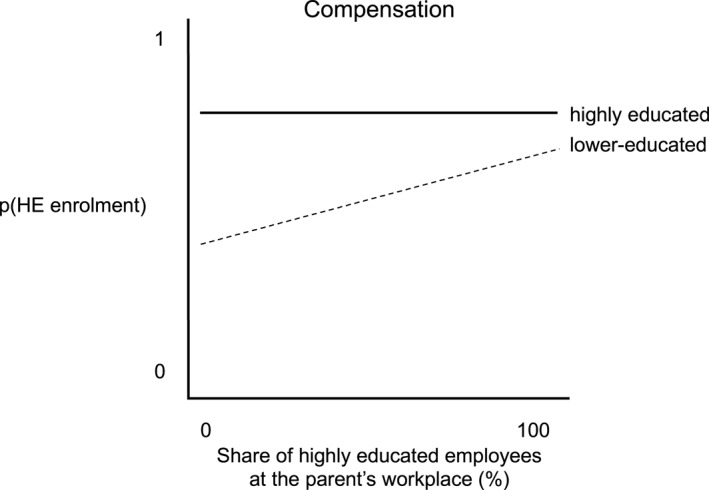
“Compensation”‐hypothesis.

Altogether, studies show that diverse work environments are associated with better career outcomes particularly for individuals from low socioeconomic backgrounds. Furthermore, it is known that parents' connections to highly educated individuals, such as highly educated extended family members, are positively associated with their children's educational attainment in lower‐educated families. Our contribution in this paper is to bridge these two literature by examining the compensatory role of diverse parental workplaces in shaping children's educational attainment. In our “Compensation”‐hypothesis (Figure 1), we assume that parental workplace educational composition is positively associated with children's higher education enrolment in lower‐educated families, as for them, connections to highly educated colleagues may break down the information barriers and thus be beneficial for educational mobility. In other words, we assume that the lack of informal knowledge about the higher education system, which is embedded in a socially stratified manner in parental networks, could potentially be compensated for through the information flows of workplace weak ties. These co‐worker ties can contribute to families’ educational decision‐making, as these types of ties often connect people who have access to different kinds of information, as they are embedded in distinct groups (Granovetter 1973).

## 3 Data and Methods

To study this topic, we used administrative full‐population register data obtained from Statistics Finland. In addition to basic information (FOLK basic data), the datasets used included educational enrolment registers, educational application registers, educational degree registers (FOLK degree/qualification), income registers (FOLK income), and employment registers (FOLK employment).

First, we chose all citizens permanently living in Finland at age 15 who were born in the years 1989–1993 and who were alive when they turned 25 (*N* = 329,788). All individuals were linked to their parents (207 individuals without any information on either parent at age 15 were excluded from the sample). We restricted the sample to individuals who had a mother, or father employed at a workplace with at least five employees during the year we observed parental employment. We focus on each parent's workplace composition separately, referring to these two distinct groups as the “paternal workplace” (*N* = 192,082, 58% of all) and the “maternal workplace” (*N* = 233,165, 71% of all). Additionally, we excluded those few participants with missing information in key variables (paternal workplace: *N* = 183,804, 56% of all; maternal workplace: *N* = 222,913, 68% of all). Appendix presents the summary statistics of the full cohort (Table A1).

Our dependent variable is enrolment in any type of higher education (yes/no) when the individual was 19–25 years old and is thus a binary outcome. Studying in any type of higher education institution in Finland at ISCED levels 6 (Bachelor's), 7 (Master's), and 8 (Doctoral) is considered enrolment in higher education. Statistics Finland has gathered this information from educational institutions and has data on all students who have the right to study and who have started their studies at a higher education institution in Finland (either a university or a university of applied sciences/polytechnic).

Our first main independent variable is parental educational level. This variable contains four distinct groups: 1) both parents have less than a higher education degree; 2) father has a higher education degree and mother has less than a higher education degree; 3) mother has a higher education degree and father has less than a higher education degree; and 4) both parents have higher education degrees. Higher education degrees are also classified according to ISCED levels 6 (Bachelor's), 7 (Master's), and 8 (Doctoral), and refer to completed qualifications. Less than a higher education degree includes all other ISCED levels as well as parents whose education level is unknown. Information on parents' education levels is derived from degree registers from the year the child turned 15.

The second main independent variable is a relative measure of the parent's workplace educational composition (share of highly educated employees in the workplace, 0%–100%). Using the full population data and unique workplace establishment IDs from the employment registers, we calculated this by obtaining information on all employees at the workplace and their educational levels. Workplace is measured primarily based on the longest employment relationship during the year, and secondarily based on the employment relationship in the final week of the year. Additionally, workplace is identified primarily by the encrypted establishment code; if that is unavailable, the encrypted enterprise code is used instead. The variable was manually constructed annually by clustering all employees into workplaces using full‐population employment registers, and then calculating the proportion of highly educated employees by dividing their number by the total number of employees at each workplace. As for parents, higher education degrees for employees are also classified according to ISCED levels 6 (Bachelor's), 7 (Master's), and 8 (Doctoral), and refer to completed qualifications. Less than a higher education degree includes all other ISCED levels as well as employees whose education level is unknown. Information about the parents' workplaces for the main analyses was obtained from the year the child turned 18, whereas in the appendix, we also present results for age 15, as many other key variables are derived from that year (Figure A8). We use workplace educational composition mainly as a continuous variable, but show the main results using a categorical measure in the Appendix (Figures A2 and A3).

Our control variables included parent's monthly taxable individual earnings based on the 2016 euros (annual earnings divided by 12, with missing values replaced by zero), parent's workplace sector (private, public), child's teacher‐given grades at the end of comprehensive school (ranging from 4 to 10 from lowest to highest), area of residence when the individual was 15 years old (urban municipalities, semi‐urban municipalities, and rural municipalities), and child's sex (female, male). Parent's earnings are derived from the income registers and were controlled to focus on the role of the workplace composition rather than the parent's position in the workplace. Parental workplace sector is derived from the employment registers and classified based on the ownership accordingly: private domestic and foreign ownership are classified as private, whereas state, municipality, and the region of Åland are classified as public. Parental workplace sector and parental earnings are always measured in the same year as the workplace educational composition. Teacher‐given grades from comprehensive school, the only measure of school performance available for the entire age cohort in the Finnish case, were controlled in the models, as we are mainly interested in educational choices net of educational performance. This variable is derived from the upper secondary application registers from years 2004–2010 and excludes the few individuals who did not apply to any upper secondary school (see the missingness above and in Table A1). If an individual had multiple records from different years, the highest grade point average was selected. The area of residence was controlled for as a possible confounder, as lower‐educated parents living in urban municipalities are more likely to work around highly educated co‐workers compared to lower‐educated parents living in rural municipalities, and moreover, children living in urban municipalities are more likely to enrol in higher education. This variable is derived from basic registers (FOLK basic) and is based on Statistics Finland's ready‐made classification.

**FIGURE A1:**
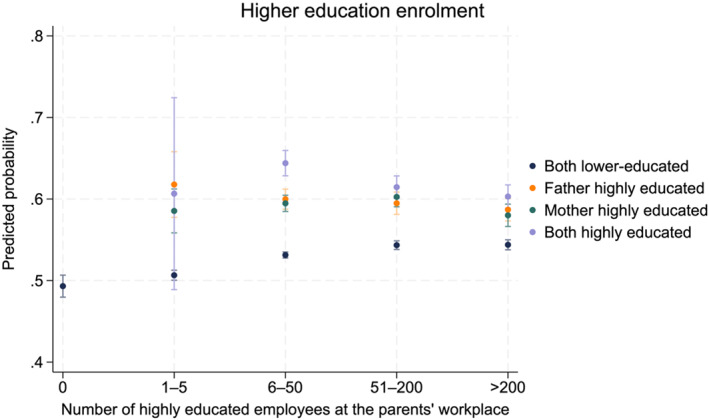
Higher education enrolment according to parental education and the number of highly educated employees at the parents' workplace (sample restricted to those who had both a mother and a father employed at a workplace with at least 5 employees when the child was 18 years old; *N* = 143,123). The model controls for area of residence, comprehensive school GPA, father's earnings, mother's earnings, father's workplace sector, mother's workplace sector, and sex. The estimates have a 95% confidence interval. Logistic regression.

**FIGURE A2:**
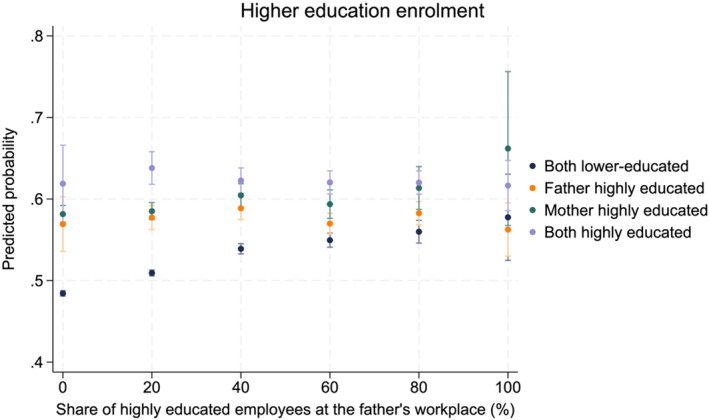
Higher education enrolment according to parental education and father's workplace educational composition as a categorical variable (paternal workplace: *N* = 183,804). The model controls for area of residence, comprehensive school GPA, father's earnings, father's workplace sector, and sex. The estimates have a 95% confidence interval. Logistic regression.

**FIGURE A3:**
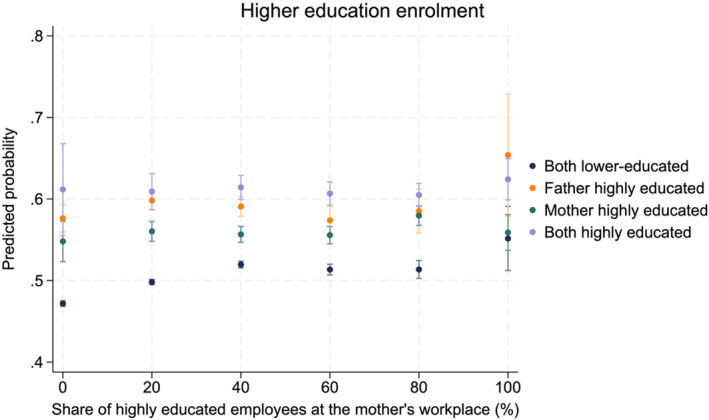
Higher education enrolment according to parental education and mother's workplace educational composition as a categorical variable (maternal workplace: *N* = 222,913). The model controls for area of residence, comprehensive school GPA, mother's earnings, mother's workplace sector, and sex. The estimates have a 95% confidence interval. Logistic regression.

Throughout the study, our unit of analysis is the child, and results are shown separately for fathers' and mothers' workplaces, as the relative measure of workplace educational composition is specific to each parent. Thus, results are displayed separately for two samples: one based on mothers and one on fathers. A child may be present in only one or in both samples. In addition to the relative measure of workplace educational composition—which requires separating the sample into two—the appendix also presents our main results using an absolute measure: the number of both parents' co‐workers, combining the two samples (Figure A1).

We begin by presenting descriptive statistics for all variables included in the study. Next, we show the distribution of highly educated employees across parental education groups. This is followed by displaying the shares of higher education enrolment across the workplace educational composition distribution by parental education groups.

As our research question involves individuals choosing between two alternatives, and our dependent variable is therefore dichotomous, we analysed the data using logistic regression. This is a well‐established technique for analysing dichotomous outcomes involving choice (Aldrich and Nelson 1984), particularly in the context of educational decisions. All our estimates from logistic regressions are converted into marginal effects. Models demonstrating the stepwise inclusion of control variables are presented in the Appendix (Tables A6 and A7). In these models, control variables were added sequentially, one at a time. The final models include, first, all independent variables from the main models (no interaction effects included), and second, all independent variables from the main models along with workplace industry and parental class (EGP), which we use to study the robustness of our results. All these estimates are converted into average marginal effects due to their ease of interpretation and comparability of effect sizes across models (Mood 2010).

In our main models presented in the text, we included an interaction term for parental education and workplace educational composition. This allowed us to examine the main focus of the study: how the strength of the association between parental education and children's educational outcomes varies across the distribution of workplace educational composition. We calculated predictive margins of higher education enrolment over parental education groups at fixed values of workplace educational composition. We display these predicted probabilities visually, as graphical representation is essential when interpreting interaction effects in logistic regression models, especially those including one continuous and one nominal independent variable (Mize 2019).

## 4 The Institutional Context of the Study: Overview of the Finnish Educational System

The Finnish educational system comprises three levels. The first level is a compulsory comprehensive school, which lasts 9 years (starting at the age of seven) and provides a basic level of education for all children. The first important educational choices are made near the end of comprehensive school at the age of 16, when students choose their upper secondary school track. There are two main tracks to choose from: general or vocational upper secondary school and the intake is based on teacher‐given compulsory school grades. General upper secondary schools focus on teaching general academic skills, whereas field‐specific vocational upper secondary schools teach students about various trades and prepare them to enter the workforce as qualified workers. All upper secondary‐level schools last for 3–4 years and provide the students with the eligibility to apply to any higher education institution.

The Finnish higher education system has a dual structure and comprises academically oriented universities and vocationally oriented polytechnics (universities of applied science), both of which provide teaching and research in almost all fields of study. During our study period, access to higher education was mainly based on entrance exams, grades from matriculation exams (general upper secondary exit exams), and/or grades from vocational upper secondary schools (accessing polytechnics). Both institutions, universities and polytechnics, provide first‐ and second‐cycle university degrees. At universities, most basic‐level study programmes include both first‐ and second‐cycle degrees, and most university graduates are master's‐level graduates. In the polytechnics, unlike universities, most basic‐level study programmes include only the first‐cycle degree, and most graduates are bachelor's‐level graduates.

Intergenerational educational inequality has been internationally compared at a very low level in Finland (Pfeffer 2008). Recently, the intergenerational educational inequality in Finland has increased, reaching average European levels (Härkönen and Sirniö 2020). The upper secondary school track is the most important choice in the Finnish educational system concerning intergenerational educational persistence (Härkönen and Sirniö 2020). At the higher levels of education, Finland has an extremely selective higher education system (OECD 2019), and enrolment in higher education is socially stratified (Heiskala, Erola, and Kilpi‐Jakonen 2021), emphasising access to higher education as an important research topic of intergenerational inequality in Finland. Due to its (socially stratified) selectivity, and because we are interested in how the higher education levels of parents' co‐workers moderate the association between parents' and children's education levels, we focus on higher education enrolment rather than upper secondary school choice. However, it is worth noting that part of this association may be mediated through upper secondary school choice, as decisions at both the upper secondary and tertiary levels tend to be path‐dependent (e.g. Heiskala, Erola, and McMullin 2021; Härkönen and Sirniö 2020).

## 5 Results

Table 1 presents the summary statistics of the variables. Approximately half of the youngsters entered higher education by the age of 25. Regarding the parent generation, three out of four families had no higher education degrees. Thus, in approximately 25% of the families, either parent was highly educated. On average, 24% of the employees in workplaces where the fathers worked and 28% of the employees in workplaces where the mothers worked had a higher education degree. While there are no substantial differences between the two samples based on mothers and fathers in terms of any of the child's variables, it is worth noting that fathers more often work in the private sector and, on average, have higher earnings compared to mothers.

**TABLE 1:** Descriptive statistics of the samples: proportions of categorical variables, means, and standard deviations of continuous variables.

	Paternal workplace (*N* = 183,804)		Maternal workplace (*N* = 222,913)	
	Mean (%)	SD	Mean (%)	SD
Higher education enrolment				
Yes	0.53		0.51	
No	0.47		0.49	
Parental education				
Both lower‐educated	0.74		0.75	
Father highly educated	0.08		0.07	
Mother highly educated	0.09		0.11	
Both highly educated	0.09		0.07	
Highly educated employees at the parent's workplace	0.24	0.24	0.28	0.24
Workplace sector				
Private	0.72		0.44	
Public	0.28		0.56	
Area of living				
Urban municipalities	0.64		0.63	
Semi‐urban municipalities	0.19		0.18	
Rural municipalities	0.17		0.19	
Comprehensive school grade point average (4–10)	7.8	1.1	7.7	1.1
Parent's monthly earnings (€)	4432.2	3661.2	3019.3	1555.1
Sex				
Female	0.49		0.49	
Male	0.51		0.51	

**FIGURE 2:**
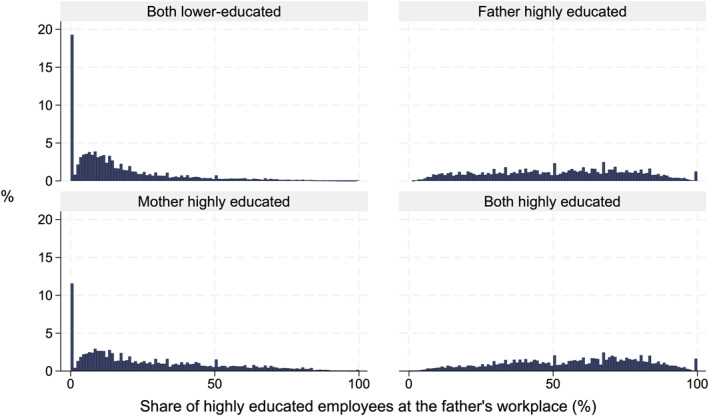
Distribution of highly educated employees at the father's workplace, based on parental education (paternal workplace: *N* = 183,804).

**FIGURE 3:**
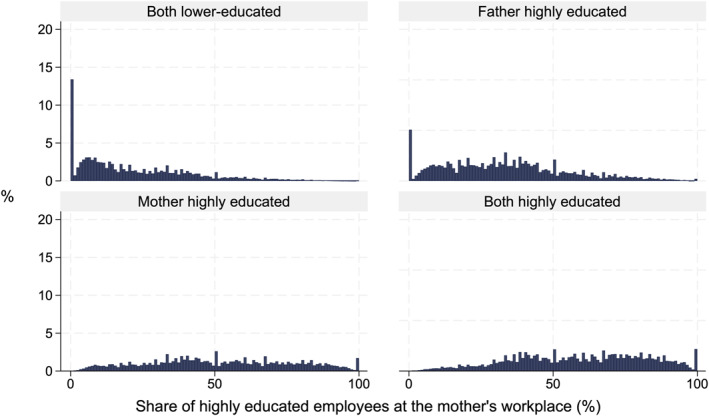
Distribution of highly educated employees at the mother's workplace, based on parental education (maternal workplace: *N* = 222,913).

To compare the differences in parents' workplace educational compositions, the histograms in Figures 2 and 3 display the distribution of the share of highly educated employees in the workplace for the parental education groups. Most parents without a higher education degree were employed in workplaces with very few or no highly educated employees (Figures 2 and 3). For example, almost one fifth of lower‐educated fathers who do not have a highly educated spouse work among only lower‐educated employees. In contrast, as can be expected, highly educated parents usually work in workplaces with at least some highly educated employees.

To study the plain association between parents and children's education using the whole cohort (*N* = 329,788), we examined the proportions of higher education enrolment for the four groups based on parents' education. As can be expected, children with highly educated parents and especially two highly educated parents were much more likely to enrol in higher education compared to children with lower‐educated parents. The majority (82%) of those with two highly educated parents enroled in higher education by the age of 25 years, whereas less than half (40%) of those with two lower‐educated parents did so. Approximately two out of three individuals from families where the father is highly educated and the mother is lower‐educated (70%), as well as from families where the mother is highly educated and the father is lower‐educated (66%), enroled in higher education. These results agree with previous studies from Finland (e.g. Heiskala, Erola, and Kilpi‐Jakonen 2021) and underline the social stratification of higher education enrolment. Next, we study how this association varies over the parent's workplace educational composition.

**FIGURE 4:**
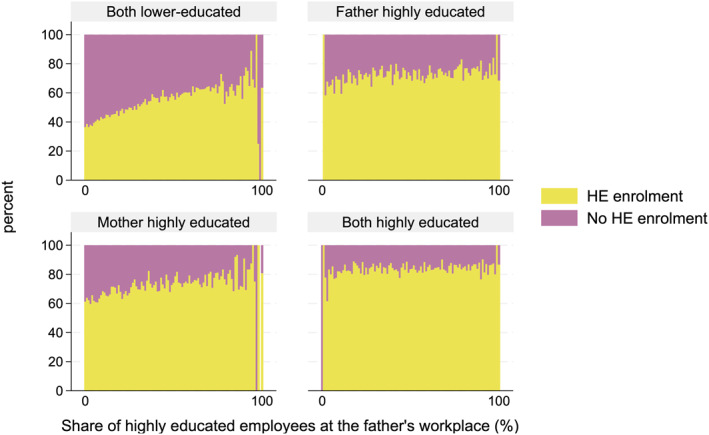
Shares of enrolment in higher education over the father's workplace educational composition, based on parental education (paternal workplace: *N* = 183,804).

**FIGURE 5:**
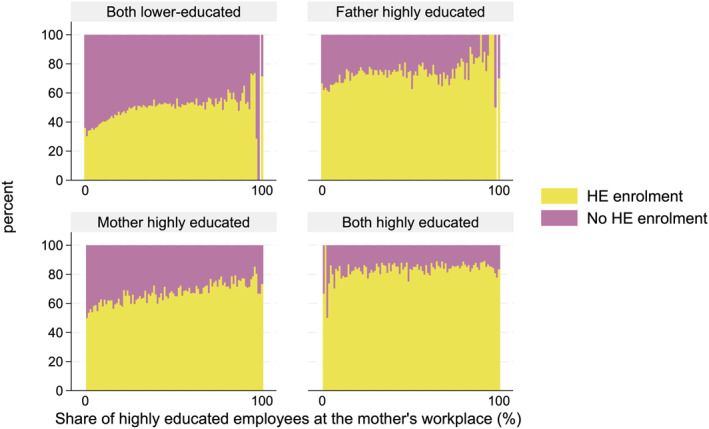
Shares of enrolment in higher education over the mother's workplace educational composition, based on parental education (maternal workplace: *N* = 222,913).

Figures 4 and 5 present the shares of higher education enrolments over the parent's workplace educational composition for four parental education groups. As described above, those with highly educated parents enroled in higher education more often than those without such parents. The figures highlight the substantial difference between the four parental education groups when we consider workplace educational composition; Figures 4 and 5 reveal that, for those with highly educated parents (with one or especially two), the share of highly educated employees in the parent's workplace plays a minor role in children's higher education enrolment. However, particularly for children with two lower‐educated parents, workplace educational composition plays an essential role: the larger the share of highly educated employees at the parent's workplace, the larger the share of higher education enrolment among such parents' children. The proportion of those enrolling in higher education among children with lower‐educated parents is around 40% for those whose parents do not have highly educated co‐workers (or very few such co‐workers), whereas this proportion increases to over 60% for those whose parents are working mainly among highly educated colleagues. Our results show that the association between workplace educational composition and higher education enrolment for children from lower‐educated families is rather linear, and even a small share of highly educated employees is associated with an increased probability of enrolment in higher education for lower‐educated families. The few outliers on the right end are due to the small number of observations for workplaces where nearly all co‐workers are highly educated, particularly among lower‐educated parents.

**FIGURE 6:**
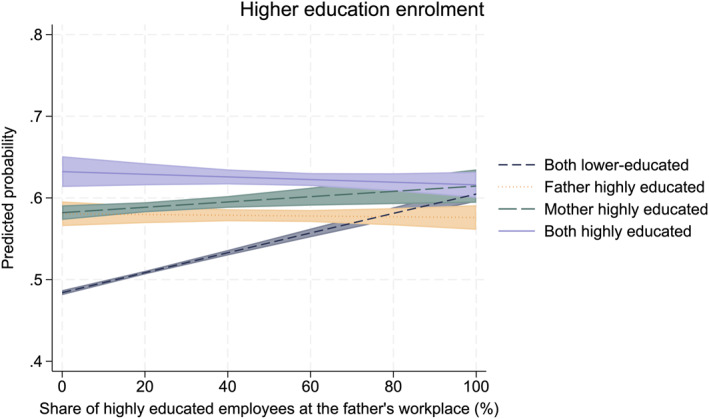
Higher education enrolment according to parental education and father's workplace educational composition (paternal workplace: *N* = 183,804). The model controls for area of residence, comprehensive school GPA, father's earnings, father's workplace sector, and sex. The estimates have a 95% confidence interval. Logistic regression.

**FIGURE 7:**
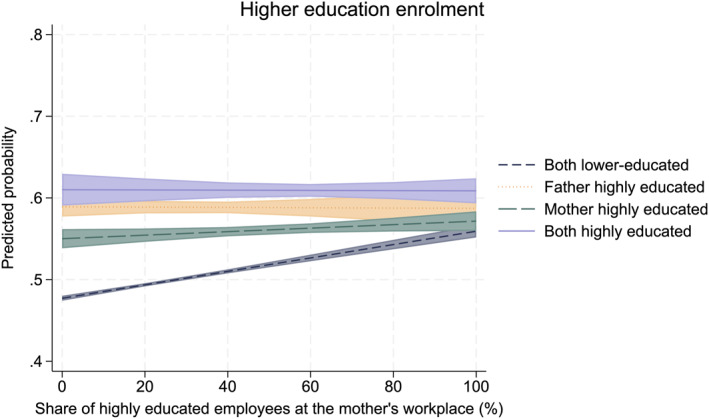
Higher education enrolment according to parental education and mother's workplace educational composition (maternal workplace: *N* = 222,913). The model controls for area of residence, comprehensive school GPA, mother's earnings, mother's workplace sector, and sex. The estimates have a 95% confidence interval. Logistic regression.

In Figures 6 and 7, we display the predicted probability of higher education enrolment for four distinct parental education groups at fixed values of workplace educational composition (see Tables A6 and A7 in the Appendix for stepwise regression models excluding the interaction term). These figures show that the association observed in the descriptive figures holds even after accounting for parents' earnings, parents' workplace sector, the family's area of residence, the child's school grades, and the child's sex.

The social origin gap in higher education enrolment between children of both highly educated and those of both lower‐educated parents in higher education enrolment is the largest (over 10% points) among those with a parent working around non‐highly educated co‐workers (Figures 6 and 7). This gap disappears in the case of fathers and substantially reduces in the case of mothers as the share of highly educated employees increases. Only among families in which both parents are lower‐educated is the share of highly educated employees at the parent's workplace substantially and positively associated with children's higher education enrolment. For other parental education groups, the workplace educational composition does not moderate the association between parental and children's education. In short, children from lower‐educated families are more likely to enrol in higher education if their parents are working around highly educated co‐workers, as shown descriptively in Figures 4 and 5. Thus, our results support our compensation hypothesis: for lower‐educated families, having a parent who works with highly educated colleagues increases the probability that their children will enrol in higher education.

One limitation of our study is that we cannot observe selection into workplaces. For instance, lower‐educated parents who work alongside highly educated colleagues may differ in important ways from those who work primarily with other lower‐educated employees. This selection could also be related to their children's likelihood of pursuing higher education. However, we are able to observe the industry in which parents are employed. We use this information descriptively to examine whether workplaces differ substantially based on their educational composition. In the Appendix (Tables A2 and A4), we present the 10 most common industries across four categories of workplace educational composition: 0%, 1%–25%, 26%–50%, and more than 50% of employees with higher education degrees. As expected, workplaces vary by educational composition, and these patterns also differ between mothers and fathers. Among fathers, the most common industries in workplaces with no highly educated employees are transport and construction. For mothers, these are retail and food service. In contrast, educational institutions are the most common workplaces where more than half of employees hold higher education degrees. We also identify the 10 most common industries specifically for lower‐educated parents who work alongside highly educated colleagues (Tables A3 and A5). Lower‐educated fathers in such settings are most frequently employed in engineering‐related fields, while lower‐educated mothers are most commonly found in educational institutions.

Despite the fact that lower‐educated mothers and fathers work in very different industries compared to each other, our main findings were consistent across these groups, as illustrated in Figures 6 and 7. Thus, the compensatory advantage of working in highly educated environments extends across a broad range of industries. Therefore, we assume that our results are not solely driven by selection effects—at least not with respect to the industry of employment.

### 5.1 Robustness Checks

We performed additional analyses to verify the robustness of our results. First, we stratified the sample based on workplace size (less than 10 employees/10–49 employees/50–249 employees/more than 250 employees) to see whether employees' educational composition moderates the association between parents' and children's education only in smaller companies where co‐workers are more likely to know each other. However, we found no substantial differences in the results based on workplace size (Figures A6 and A7). Second, we measured parents' employment at different time points: when the children were aged 10, 15, or 20 years (in addition to age 18 as shown in the main analyses) as well as taking the mean and the maximum of the share of highly educated co‐workers at the parent's workplace when the child was 10–25 and 13–19 years old. In these cases as well, our results remained consistent (see Figure A8 for results using workplace educational composition measured when child was 15 years old; additional figures are available upon request). Third, we stratified the sample by workplace sector (private/public) and found similar results (Figure A5).

**FIGURE A4:**
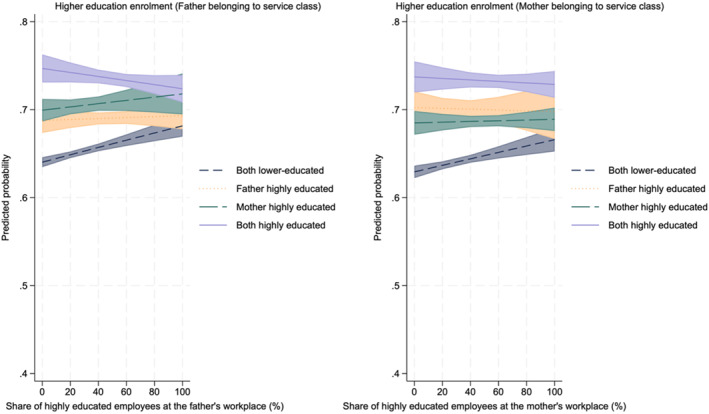
Higher education enrolment for individuals with a parent in the service class, by parental education and parent's workplace educational composition. The models control for area of residence, comprehensive school GPA, parent's earnings, parent's workplace sector, and sex. The estimates have a 95% confidence interval. Logistic regression. Sample sizes: left panel *N* = 87,257; right panel *N* = 76,918.

**FIGURE A5:**
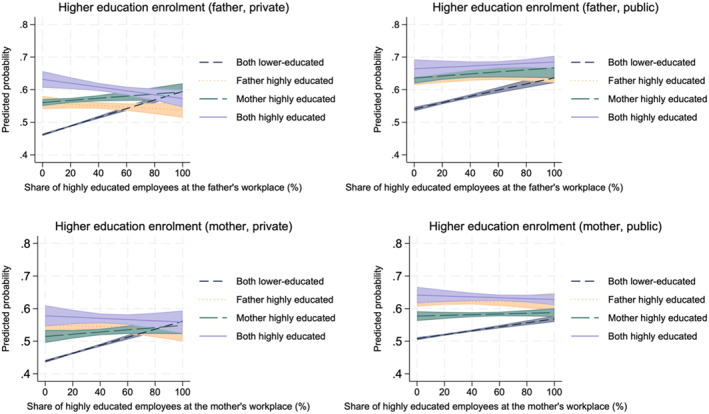
Higher education enrolment according to parental education and parent's workplace educational composition, stratified by parent's workplace sector. The models control for area of residence, comprehensive school GPA, parent's earnings, and sex. The estimates have a 95% confidence interval. Logistic regression. Sample sizes: upper‐left panel *N* = 131,916; upper‐right panel *N* = 51,919; lower‐left panel *N* = 98,503; lower‐right panel *N* = 124,511.

**FIGURE A6:**
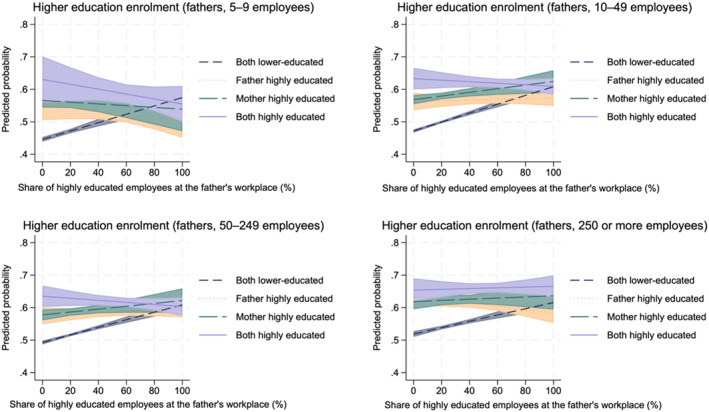
Higher education enrolment according to parental education and father's workplace educational composition, stratified by father's workplace size. The models control for area of residence, comprehensive school GPA, father's earnings, father's workplace sector, and sex. The estimates have a 95% confidence interval. Logistic regression. Sample sizes: upper‐left panel *N* = 22,421; upper‐right panel *N* = 66,274; lower‐left panel *N* = 58,133; lower‐right panel *N* = 37,007.

**FIGURE A7:**
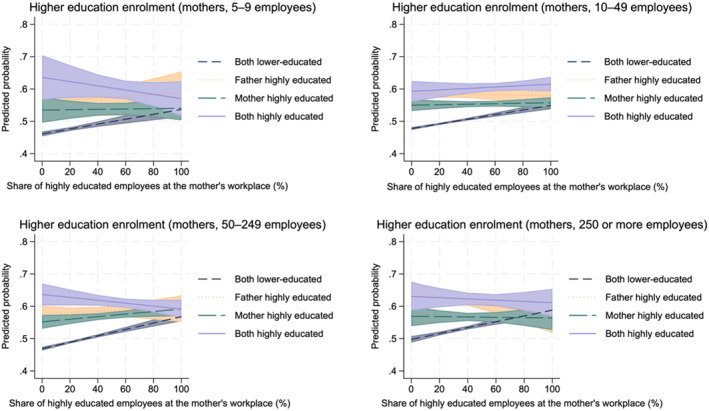
Higher education enrolment according to parental education and mother's workplace educational composition, stratified by mother's workplace size. The models control for area of residence, comprehensive school GPA, mother's earnings, mother's workplace sector, and sex. The estimates have a 95% confidence interval. Logistic regression. Sample sizes: upper‐left panel *N* = 25,116; upper‐right panel *N* = 93,817; lower‐left panel *N* = 64,828; lower‐right panel *N* = 39,253.

**FIGURE A8:**
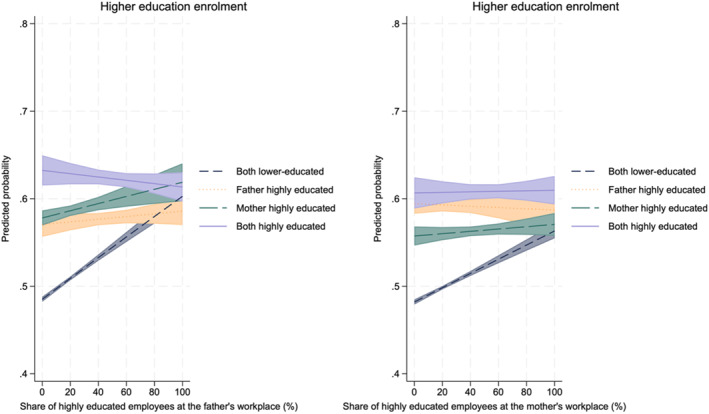
Higher education enrolment according to parental education and parent's workplace educational composition when the child is 15 years old. The models control for area of residence, comprehensive school GPA, parent's earnings, parent's workplace sector, and sex. The estimates have a 95% confidence interval. Logistic regression. Sample sizes: left panel *N* = 191,705; right panel *N* = 219,067.

Lastly, we explored whether the parent's own socioeconomic class position, measured using the EGP (Erikson‐Goldthorpe‐Portocarero) class scheme, could explain the associations we found. First, lower‐educated parents working with highly educated colleagues belonged to the service class much more often compared to the lower‐educated parents whose co‐workers were not highly educated. Among highly educated parents, most workers belonged to the service class, independent of the workplace's educational composition. To determine whether there was an increase in the probability of higher education enrolment among children of lower‐educated parents working around highly educated colleagues only because these parents belonged to a high socioeconomic class by themselves, we ran the main analyses among parents belonging to the service class. Even among service‐class parents, the compensatory advantage of highly educated co‐workers for children from lower‐educated families can still be observed (Figure A4).

## 6 Discussion and Conclusions

Informal knowledge about the educational system is important for navigating it especially at higher levels of education (Forster and van de Werfhorst 2020). Class‐related information barriers limit choices and understanding—especially from a lower‐class perspective— regarding higher education. Our aim in this study was to explore whether educationally diverse parental workplaces, in which less and highly educated parents are likely to meet, could reduce social origin differences in children's educational attainment. Previous studies have shown that connections to highly educated adults, such as extended family members, can compensate for low parental resources (Jæger 2012; Erola et al. 2018; Lehti et al. 2019). We studied whether non‐kin connections, which are less explored in this regard, operate in a similar way. Using high‐quality Finnish full population register data, we were able to show that having a parent who was working around highly educated co‐workers was beneficial, in terms of higher education enrolment, for children from lower‐educated families.

Our results show that parental weak ties (Granovetter 1973) can compensate for a lack of resources in the immediate family and reduce the social origin gap in higher education enrolment. These weak ties may play a role in breaking down existing biases and information barriers regarding parents' contributions to families' educational decision‐making processes. Information shared through workplace networks may shape knowledge on various topics, including the prospects of different educational paths and potential career outcomes for children. However, it is worth noting that weak ties are usually captured using survey or network data, whereas measuring them with register data is less common (for an example of capturing weak ties using register data, see von Essen and Smith 2023). Whether the differences we find are due to actual (strong or weak) ties between co‐workers and whether interactions between workmates include conversations about their children's education or the higher education system in general, we cannot determine with register data. Parents may also, for example, pass on occupation‐specific capital to their children (Jonsson et al. 2009), which could partly explain our results. Overall, our findings underscore the role of parental workplaces in enhancing low social origin children's educational outcomes, even if the precise mechanisms driving these processes remain open to debate.

The importance of workplaces is not surprising, as working‐age adults spend a relatively large share of their day in the workplace and are likely to form weak ties, essential for information flow, with co‐workers. Previous studies have shown how co‐worker networks play an important role especially in individuals' labour market transitions (Glitz 2017; Baranowska‐Rataj et al. 2023). It is also worth mentioning that workplaces seem to be rather segregated environments in terms of employee educational levels. Furthermore, previous studies have shown that individuals usually make connections with people who are similar to themselves (McPherson et al. 2001). However, being simply exposed to different types of people is essential in building economic connectedness (i.e. having friends from different socioeconomic positions), which, in turn, is highly beneficial for individuals' upward social mobility (Chetty et al. 2022a). Our results add to these findings by providing an example of how diverse social spaces, where individuals are exposed to various kinds of people, can boost intergenerational social mobility by shaping families' socially stratified educational decisions.

The generalisability of these results is subject to certain limitations. The most important limitation concerns selection into workplaces. It is possible that lower‐educated parents who work alongside highly educated co‐workers differ in some unobserved way from other lower‐educated parents, which may also account for differences in their children's educational attainments. However, the consistency of our results across both paternal and maternal samples, despite lower‐educated fathers and mothers working in very different industries when surrounded by highly educated co‐workers, somewhat reduces concerns about selection bias. In addition, in our study context, it is worth noting that workplaces in Finland, as in other Nordic countries, generally have flat organizational structures and hierarchies (Andreasson and Lundqvist 2018), which might positively affect information flows and connections between co‐workers. Therefore, the associations we found may be stronger than those found in other countries.

Despite these limitations, this study enhances our understanding of the significance of parental weak ties and diverse environments, such as parental workplaces. Addressing and potentially mitigating socioeconomic segregation in institutional settings like workplaces are crucial steps towards boosting intergenerational social mobility.

## Ethics Statement

Research based solely on register data does not require the consent of the individuals investigated or ethical approval. Good scientific practice and data protection regulations were followed throughout the study.

## Consent

Research based solely on register data does not require the consent of the individuals investigated or ethical approval.

## Conflicts of Interest

The authors declare no conflicts of interest

## Permission to Reproduce Material from Other Sources

The authors have Nothing to report.

## Data Availability

Statistics Finland provided permission to use the anonymized register‐based data, and access for further research and replication is granted by Statistics Finland.

## Appendix

**TABLE A1:** Descriptive statistics of the full cohort (*N* = 329,788); proportions of categorical variables; means and standard deviations of continuous variables.

	Mean (%)	SD
Higher education enrolment		
Yes	0.47	
No	0.53	
Parental education		
Both lower‐educated	0.78	
Father highly educated	0.06	
Mother highly educated	0.09	
Both highly educated	0.06	
Area of living		
Urban municipalities	0.60	
Semi‐urban municipalities	0.19	
Rural municipalities	0.21	
Comprehensive school grade point average (4–10)	7.7	1.1
Missing for	0.05	
Father's monthly earnings (€)	3379.5	3370.6
Mother's monthly earnings (€)	2579.7	1707.3
Sex		
Female	0.49	
Male	0.51	

**TABLE A2:** Ten most common industries (4‐digit NACE Rev. 2) for fathers, by the educational composition of the father's workplace.

% of this subgroup	% of all fathers in the sample	Industry
**Subgroup: share of highly educated employees at the father's workplace: > 50%**		
10.16	1.77	Tertiary education
9.84	3.12	Engineering activities and related technical consultancy
6.92	1.19	General secondary education
6.48	1.94	Technical and vocational secondary education
6.3	1.37	Manufacture of communication equipment
6.3	1.19	Primary education
3.66	1.06	Computer programing activities
3.6	0.74	Other research and experimental development on natural sciences and engineering
2.65	1.12	General public administration activities
2.04	1.65	Defence activities
**Subgroup: share of highly educated employees at the father's workplace: 26%–50%**		
7.35	1.84	Hospital activities
4.51	1.65	Defence activities
4.19	1.94	Technical and vocational secondary education
3.01	3.12	Engineering activities and related technical consultancy
2.82	0.94	Activities of religious organisations
2.50	4.04	Construction of residential and non‐residential buildings
2.17	1.06	Computer programing activities
2.08	0.67	Other monetary intermediation
2.06	1.12	General public administration activities
1.96	0.69	Computer facilities management activities
**Subgroup: share of highly educated employees at the father's workplace: 1%–25%**		
4.79	4.04	Construction of residential and non‐residential buildings
3.55	1.89	Manufacture of paper and paperboard
2.27	1.45	Public order and safety activities
2.04	2.54	Freight transport by road
1.97	1.21	Machining
1.91	1.44	Plumbing, heat and air‐conditioning installation
1.85	1.17	Postal activities under universal service obligation
1.67	1.08	Repair of machinery
1.65	0.86	Manufacture of basic iron and steel and of ferro‐alloys
1.53	0.97	Manufacture of other builders' carpentry and joinery
**Subgroup: share of highly educated employees at the father's workplace: 0%**		
9.86	2.54	Freight transport by road
7.64	4.04	Construction of residential and non‐residential buildings
3.67	1.19	Combined facilities support activities
3.18	1.09	Maintenance and repair of motor vehicles
3.05	0.99	Other specialised construction activities n.e.c.
2.90	1.44	Plumbing, heat and air‐conditioning installation
2.56	0.87	Site preparation
2.00	1.07	Electrical installation
1.72	0.67	Logging
1.72	0.38	Retail trade of motor vehicle parts and accessories

**TABLE A3:** Ten most common industries (4‐digit NACE Rev. 2) for lower‐educated fathers, by the educational composition of the father's workplace.

% of lower‐educated fathers in this subgroup	Industry
**Subgroup: share of highly educated employees at the father's workplace: > 50%**	
15.13	Engineering activities and related technical consultancy
8.80	Technical and vocational secondary education
7.73	Manufacture of communication equipment
4.96	Tertiary education
4.40	Computer programing activities
4.19	Primary education
2.99	General secondary education
2.41	Manufacture of machinery for paper and paperboard production
2.38	Other research and experimental development on natural sciences and engineering
2.24	Computer facilities management activities
**Subgroup: share of highly educated employees at the father's workplace: 26%–50%**	
5.56	Hospital activities
4.69	Defence activities
4.24	Technical and vocational secondary education
3.24	Engineering activities and related technical consultancy
3.13	Construction of residential and non‐residential buildings
2.25	Wholesale of other machinery and equipment
2.12	Computer programing activities
1.94	Computer facilities management activities
1.92	Wired telecommunications activities
1.85	Manufacture of lifting and handling equipment
**Subgroup: share of highly educated employees at the father's workplace: 1%–25%**	
4.93	Construction of residential and non‐residential buildings
3.46	Manufacture of paper and paperboard
2.18	Public order and safety activities
2.12	Freight transport by road
2.03	Machining
1.98	Plumbing, heat and air‐conditioning installation
1.87	Postal activities under universal service obligation
1.70	Repair of machinery
1.60	Manufacture of basic iron and steel and of ferro‐alloys
1.58	Manufacture of other builders' carpentry and joinery
**Subgroup: share of highly educated employees at the father's workplace: 0%**	
9.85	Freight transport by road
7.64	Construction of residential and non‐residential buildings
3.67	Combined facilities support activities
3.18	Maintenance and repair of motor vehicles
3.05	Other specialised construction activities n.e.c.
2.90	Plumbing, heat and air‐conditioning installation
2.56	Site preparation
2.00	Electrical installation
1.72	Logging
1.72	Retail trade of motor vehicle parts and accessories

**TABLE A4:** Ten most common industries (4‐digit NACE Rev. 2) for mothers, by the educational composition of the mother's workplace.

% of this subgroup	% of all mothers in the sample	Industry
**Subgroup: share of highly educated employees at the mother's workplace: > 50%**		
18.01	4.04	Primary education
13.06	2.62	General secondary education
9.54	1.88	Tertiary education
7.05	2.23	Technical and vocational secondary education
3.48	9.36	Hospital activities
3.42	2.44	General public administration activities
3.38	1.81	Regulation of the activities of providing health care, education, cultural services and other social services, excluding social security
2.46	6.37	General medical practice activities
2.38	1.13	Engineering activities and related technical consultancy
2.38	0.56	Other research and experimental development on natural sciences and engineering
**Subgroup: share of highly educated employees at the mother's workplace: 26%–50%**		
23.36	9.36	Hospital activities
9.32	6.37	General medical practice activities
7.65	7.29	Child day‐care activities
3.83	2.08	Other monetary intermediation
3.13	2.44	General public administration activities
2.84	1.55	Activities of religious organisations
2.70	2.23	Technical and vocational secondary education
2.45	1.81	Regulation of the activities of providing health care, education, cultural services and other social services, excluding social security
2.20	4.04	Primary education
1.80	0.87	Specialist medical practice activities
**Subgroup: share of highly educated employees at the mother's workplace: 1%–25%**		
10.10	7.29	Child day‐care activities
7.21	6.37	General medical practice activities
5.44	2.63	Residential nursing care activities
5.40	3.02	Social work activities without accommodation for the elderly and disabled
5.15	2.81	Residential care activities for the elderly and disabled
4.59	9.36	Hospital activities
3.45	2.04	General cleaning of buildings
2.61	1.38	Other retail sale in non‐specialised stores
2.52	1.46	Residential care activities for mental retardation, mental health and substance abuse
2.18	1.94	Retail sale in non‐specialised stores with food, beverages or tobacco predominating
**Subgroup: share of highly educated employees at the mother's workplace: 0%**		
9.14	1.94	Retail sale in non‐specialised stores with food, beverages or tobacco predominating
7.46	1.68	Other food service activities
6.74	7.29	Child day‐care activities
5.46	2.04	General cleaning of buildings
5.27	3.02	Social work activities without accommodation for the elderly and disabled
5.11	2.81	Residential care activities for the elderly and disabled
3.79	0.8	Restaurants and mobile food service activities
3.61	0.91	Combined facilities support activities
3.04	2.63	Residential nursing care activities
2.35	0.81	Postal activities under universal service obligation

**TABLE A5:** Ten most common industries (4‐digit NACE Rev. 2) for lower‐educated mothers, by the educational composition of the mother's workplace.

% of lower‐educated mothers in this subgroup	Industry
**Subgroup: share of highly educated employees at the mother's workplace: > 50%**	
17.67	Primary education
8.45	General secondary education
6.35	Tertiary education
6.18	Technical and vocational secondary education
4.68	Hospital activities
3.80	General public administration activities
3.45	Engineering activities and related technical consultancy
3.27	Regulation of the activities of providing health care, education, cultural services and other social services, excluding social security
2.50	General medical practice activities
2.44	Other research and experimental development on natural sciences and engineering
**Subgroup: share of highly educated employees at the mother's workplace: 26%–50%**	
23.5	Hospital activities
9.02	Child day‐care activities
8.74	General medical practice activities
4.12	Other monetary intermediation
3.07	General public administration activities
2.77	Activities of religious organisations
2.39	Technical and vocational secondary education
2.26	Regulation of the activities of providing health care, education, cultural services and other social services, excluding social security
2.06	Primary education
1.69	Non‐life insurance
**Subgroup: share of highly educated employees at the mother's workplace: 1%–25%**	
10.41	Child day‐care activities
7.06	General medical practice activities
5.51	Residential nursing care activities
5.39	Social work activities without accommodation for the elderly and disabled
5.14	Residential care activities for the elderly and disabled
4.43	Hospital activities
3.58	General cleaning of buildings
2.71	Other retail sale in non‐specialised stores
2.44	Residential care activities for mental retardation, mental health and substance abuse
2.27	Retail sale in non‐specialised stores with food, beverages or tobacco predominating
**Subgroup: share of highly educated employees at the mother's workplace: 0%**	
9.14	Retail sale in non‐specialised stores with food, beverages or tobacco predominating
7.46	Other food service activities
6.74	Child day‐care activities
5.46	General cleaning of buildings
5.27	Social work activities without accommodation for the elderly and disabled
5.11	Residential care activities for the elderly and disabled
3.79	Restaurants and mobile food service activities
3.61	Combined facilities support activities
3.04	Residential nursing care activities
2.35	Postal activities under universal service obligation

**TABLE A6:** Higher education enrolment, sample by father's workplace (*N* = 183,804).

	M1	M2	M3	M4	M5	M6	M7	M8	M9	M10	M11	M12
Parental education (ref. Both lower‐educated)												
Father highly educated	0.282***		0.183***	0.183***	0.142***	0.182***	0.061***	0.181***	0.179***	0.129***	0.044***	0.032***
	(0.004)		(0.005)	(0.005)	(0.005)	(0.005)	(0.004)	(0.005)	(0.005)	(0.005)	(0.004)	(0.004)
Mother highly educated	0.243***		0.212***	0.212***	0.201***	0.212***	0.083***	0.212***	0.204***	0.189***	0.077***	0.071***
	(0.004)		(0.004)	(0.004)	(0.004)	(0.004)	(0.003)	(0.004)	(0.004)	(0.004)	(0.003)	(0.003)
Both highly educated	0.397***		0.309***	0.309***	0.267***	0.309***	0.107***	0.308***	0.307***	0.264***	0.085***	0.077***
	(0.003)		(0.004)	(0.004)	(0.005)	(0.004)	(0.004)	(0.004)	(0.004)	(0.004)	(0.004)	(0.005)
Share of highly educated employees at the father's workplace		0.005***	0.003***	0.003***	0.003***	0.003***	0.001***	0.003***	0.002***	0.002***	0.001***	< 0.001***
		(< 0.001)	(< 0.001)	(< 0.001)	(< 0.001)	(< 0.001)	(< 0.001)	(< 0.001)	(< 0.001)	(< 0.001)	(< 0.001)	(< 0.001)
Area of living (ref. Urban municipalities)												
Semi‐urban municipalities				0.006*							0.015***	0.013***
				(0.003)							(0.002)	(0.002)
Rural municipalities				0.005							0.001	0.002
				(0.003)							(0.002)	(0.003)
Father's monthly earnings					< 0.001***						< 0.001***	< 0.001***
					(< 0.001)						(< 0.001)	(< 0.001)
Sex (ref. Female)												
Male						0.094***					−0.049***	−0.047***
						(0.002)					(0.002)	(0.002)
Comprehensive school GPA							0.241***				0.246***	0.242***
							(0.001)				(0.001)	(0.001)
Father's workplace sector (ref. Private)												
Public								0.013***			0.010***	0.016***
								(0.003)			(0.002)	(0.004)
Father's workplace industry									x			x
Father’ social class (ref. EGP I‐II)												
Others (EGP III–VII)										−0.161***		−0.062***
										(0.003)		(0.002)

*Note:* Standard errors in parentheses.Average marginal effects (AME) after logistic regression. Coefficient estimates for father's workplace industry dummies (4‐digit NACE Rev. 2) are omitted from the table.

* *p* < 0.05.

** *p* < 0.01.

*** *p* < 0.001.

**TABLE A7:** Higher education enrolment, sample by mother's workplace (*N* = 222,913).

	M1	M2	M3	M4	M5	M6	M7	M8	M9	M10	M11	M12
Parental education (ref. Both lower‐educated)												
Father highly educated	0.290***		0.260***	0.262***	0.250***	0.260***	0.091***	0.260***	0.251***	0.246***	0.086***	0.081***
	(0.004)		(0.004)	(0.004)	(0.004)	(0.004)	(0.004)	(0.004)	(0.004)	(0.004)	(0.004)	(0.004)
Mother highly educated	0.237***		0.161***	0.161***	0.117***	0.160***	0.066***	0.160***	0.165***	0.120***	0.047***	0.043***
	(0.003)		(0.004)	(0.004)	(0.004)	(0.004)	(0.003)	(0.004)	(0.004)	(0.004)	(0.003)	(0.003)
Both highly educated	0.405***		0.336***	0.337***	0.282***	0.336***	0.120***	0.336***	0.339***	0.291***	0.091***	0.089***
	(0.003)		(0.004)	(0.004)	(0.005)	(0.004)	(0.004)	(0.004)	(0.004)	(0.004)	(0.004)	(0.004)
Share of highly educated employees at the mother's workplace		0.005***	0.003***	0.003***	0.002***	0.003***	0.001***	0.003***	0.002***	0.002***	0.001***	< 0.001***
		(< 0.001)	(< 0.001)	(< 0.001)	(< 0.001)	(< 0.001)	(< 0.001)	(< 0.001)	(< 0.001)	(< 0.001)	(< 0.001)	(< 0.001)
Area of living (ref. Urban municipalities)												
Semi‐urban municipalities				0.013***							0.020***	0.019***
				0.003							(0.002)	(0.002)
Rural municipalities				0.009**							0.007**	0.007***
				(0.003)							(0.002)	(0.002)
Mother's monthly earnings					< 0.001***						< 0.001***	< 0.001***
					(< 0.001)						(< 0.001)	(< 0.001)
Sex (ref. Female)												
Male						0.104***					−0.045***	−0.044***
						(0.002)					(0.002)	(0.002)
Comprehensive school GPA							0.242***				0.247***	0.244***
							(0.001)				(0.001)	(0.001)
Mother's workplace sector (ref. Private)												
Public								0.017***			0.014***	0.014***
								(0.002)			(0.002)	(0.003)
Mother's workplace industry									x			x
Mother's social class (ref. EGP I‐II)												
Others (EGP III–VII)										−0.115***		−0.037***
										(0.003)		(0.002)

*Note:* Standard errors in parentheses.Average marginal effects (AME) after logistic regression.Coefficient estimates for mother's workplace industry dummies (4‐digit NACE Rev. 2) are omitted from the table.

* *p* < 0.05.

** *p* < 0.01.

*** *p* < 0.001.
